# Exploring neurodegenerative disorders using advanced magnetic resonance imaging of the glymphatic system

**DOI:** 10.3389/fpsyt.2024.1368489

**Published:** 2024-04-08

**Authors:** Jannik Prasuhn, Jiadi Xu, Jun Hua, Peter van Zijl, Linda Knutsson

**Affiliations:** ^1^ Division of Magnetic Resonance (MR) Research, Department of Radiology, Johns Hopkins University School of Medicine, Baltimore, MD, United States; ^2^ F. M. Kirby Research Center for Functional Brain Imaging, Hugo W. Moser Research Institute at Kennedy Krieger, Baltimore, MD, United States; ^3^ Department of Neurology, University Medical Center Schleswig-Holstein, Lübeck, Germany; ^4^ Institute of Neurogenetics, University of Lübeck, Lübeck, Germany; ^5^ Center for Brain, Behavior and Metabolism, University of Lübeck, Lübeck, Germany; ^6^ Department of Neurology, Johns Hopkins University School of Medicine, Baltimore, MD, United States; ^7^ Department of Medical Radiation Physics, Lund University, Lund, Sweden

**Keywords:** neurodegeneration, Alzheimer’s disease, Parkinson’s disease, glymphatic system, neuroimaging

## Abstract

The glymphatic system, a macroscopic waste clearance system in the brain, is crucial for maintaining neural health. It facilitates the exchange of cerebrospinal and interstitial fluid, aiding the clearance of soluble proteins and metabolites and distributing essential nutrients and signaling molecules. Emerging evidence suggests a link between glymphatic dysfunction and the pathogenesis of neurodegenerative disorders, including Alzheimer’s, Parkinson’s, and Huntington’s disease. These disorders are characterized by the accumulation and propagation of misfolded or mutant proteins, a process in which the glymphatic system is likely involved. Impaired glymphatic clearance could lead to the buildup of these toxic proteins, contributing to neurodegeneration. Understanding the glymphatic system’s role in these disorders could provide insights into their pathophysiology and pave the way for new therapeutic strategies. Pharmacological enhancement of glymphatic clearance could reduce the burden of toxic proteins and slow disease progression. Neuroimaging techniques, particularly MRI-based methods, have emerged as promising tools for studying the glymphatic system *in vivo*. These techniques allow for the visualization of glymphatic flow, providing insights into its function under healthy and pathological conditions. This narrative review highlights current MRI-based methodologies, such as motion-sensitizing pulsed field gradient (PFG) based methods, as well as dynamic gadolinium-based and glucose-enhanced methodologies currently used in the study of neurodegenerative disorders.

## 1 Overview of the glymphatic system

### 1.1 Introduction

The glymphatic system is a recently discovered macroscopic waste clearance system that utilizes a unique system of perivascular channels formed by astroglial cells to promote the efficient elimination of soluble proteins and metabolites from the central nervous system (1). This system is a functional analog to the lymphatic system present in other organs. The name “glymphatic” is a portmanteau of “glial” and “lymphatic” (2). The glymphatic system is active during sleep and primarily driven by arterial pulsation and the aquaporin-4 (AQP4) s maintains health by efficiently removing waste products and distributing essential nutrients (1). The significance of the glymphatic system is stressed by its association with several neurological disorders, including neurodegenerative disorders (NDs), traumatic brain injury, and stroke, among others (3–6).

The discovery of the glymphatic system has revolutionized our understanding of central nervous system physiology. For many years, the lack of a lymphatic system in the brain led to questions about how waste products were removed (7). The discovery of the glymphatic system revealed a robust waste removal system intimately linked with the brain’s vascular system (3). This finding has also highlighted the importance of sleep for brain health, given that the glymphatic system is highly active during sleep (8). However, the physiological role of the glymphatic system is not limited to waste removal as it also plays a crucial role in distributing essential nutrients throughout the brain (1). These nutrients, including glucose, lipids, amino acids, and other molecules such as growth factors, are critical for maintaining brain health and physiological functioning (1). The distribution of nutrients via the glymphatic system is particularly important given the brain’s high metabolic demands (9). The discovery of the glymphatic system has also opened up new avenues for research. Scientists are now investigating the role of the glymphatic system in various neurological disorders with the hope of developing new therapeutic strategies (6, 10).

### 1.2 Physiology of the glymphatic system

**Figure 1:**
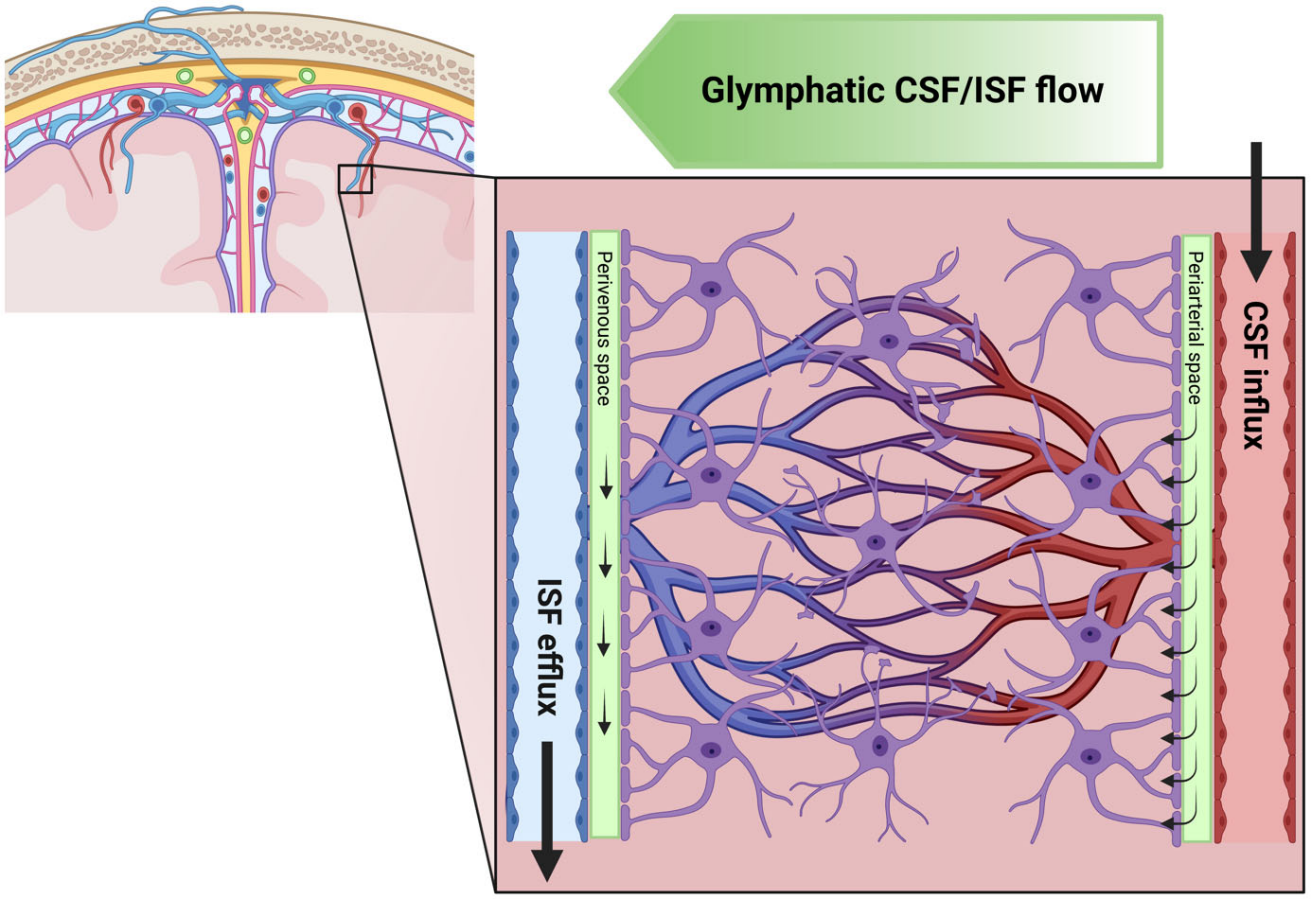
Schematic illustration of the glymphatic system. Here, we illustrate the flow of cerebrospinal fluid (CSF) and interstitial fluid (ISF) in the brain. The green gradient of the “Glymphatic CSF/ISF flow” panel depicts the direction of the waste clearance of the glymphatic system. The CSF flows from the subarachnoid space into the perivascular space of major cerebral arteries. Subsequently, the CSF flow is directed along the arteries and their branches into penetrating arteries. This figure also highlights the microscopic details of CSF flow within the brain, showing that the perivascular space runs along the entire penetrating artery (known as the Virchow–Robin space) and continues to follow the vessel as it branches into arterioles and capillaries. It further demonstrates the CSF influx into the extracellular space at every level of the perivascular space after entry to the brain parenchyma, facilitated by a polarized expression of the aquaporin 4 (AQP4) water channel towards the astrocytic endfeet lining the perivascular space. The figure also presents the concept of ISF bulk flow clearance, a phenomenon observed in animals, which could be driven by multiple factors such as CSF inflow, arterial pulsatility, hydrostatic pressure gradients between the arterial and venous perivascular spaces, and osmotic gradients. The directional flow of ISF and its solutes towards the venous perivascular space, where the fluid is taken up and drained by convection out of the brain parenchyma, is also depicted. This process effectively removes solutes accumulated during neural activity from the brain parenchyma. Created with biorender.com.

The glymphatic system plays a crucial role in maintaining the homeostasis of the neural microenvironment (1). It does so by facilitating the clearance of interstitial solutes, including for instance amyloid-beta and tau, two proteins considered pathophysiological hallmarks of Alzheimer’s disease (AD). The glymphatic system is particularly vital during sleep when the clearance of harmful waste products is up to two-fold faster than in the waking state (2). This physiological functioning of the glymphatic system is driven mainly by the interaction of various cellular and molecular components, which together facilitate the movement of cerebrospinal fluid (CSF) and interstitial fluid (ISF) within the brain (see **Figure 1**) (7). At the cellular level, the glymphatic system involves several cell types, including astrocytes, cells of the vascular system, and neurons (7). Astrocytes, with their endfeet ensheathing the brain’s blood vessels, play a crucial role in facilitating fluid movement within the glymphatic system (7). The above-stated polarization of astrocytes and the formation of endfeet form a network of perivascular tunnels, which serve as the primary pathways for fluid movement within the glymphatic system (7). Vascular cells, including endothelial cells and pericytes, also contribute to the cytoarchitecture of the glymphatic system (7). These cells play a role in maintaining the integrity of the blood-brain barrier (BBB). They are necessary for regulating cerebral blood flow (CBF), which can influence fluid movement within the glymphatic system (11). Neurons, while not directly involved in fluid motion, are believed to influence glymphatic function indirectly through their influence on the sleep-wake cycle. The sleep-wake cycle is associated with enhanced glymphatic clearance, likely due to increased interstitial space, which reduces resistance to convective fluid transport (7). At the molecular level, the function of the glymphatic system is closely tied to the expression of AQP4 water channels. These channels, primarily located on the astrocytic end feet, facilitate water movement between the CSF and ISF. The cellular distribution of AQP4 channels and the polarization of astroglia are crucial for efficient glymphatic function (12). The glymphatic system plays a significant role in the clearance of potentially harmful solutes from the brain’s interstitial space. Among these solutes, the clearance of amyloid-beta has received considerable attention (6, 13). In summary, the glymphatic system, through its cellular and molecular components, plays a crucial role in maintaining the homeostasis of the central nervous system.

### 1.3 The glymphatic system in neurodegenerative disorders

The glymphatic system has been implicated in the pathogenesis of several NDs, including AD, Parkinson’s disease (PD), and Huntington’s Disease (HD) (10, 14–16). We did not include diseases that are primarily considered to be disorders of the immune system, such as Multiple Sclerosis (MS), but might have neurodegenerative features. In AD, the most common form of dementia, the accumulation of amyloid-beta plaques in the brain is considered a key pathological feature (17). Amyloid-beta is a peptide produced in the brain as a byproduct of various cellular processes, e.g., synaptic transmission (17). Under normal conditions, amyloid-beta is cleared from the brain via several pathways, including the glymphatic system (17). However, in AD, this clearance process is believed to be impaired, leading to amyloid-beta accumulation in the brain (17). Here, studies in mice have shown that the infusion of amyloid-beta into the brain results in its clearance via the glymphatic system (18). Furthermore, amyloid-beta clearance is significantly reduced in mice lacking the AQP4 water channels, which are crucial for glymphatic function (18). AQP4 channels play a crucial role in the glymphatic system by facilitating the movement of water between CSF and ISF, thereby supporting the convective transport of solutes, including large molecules like amyloid-beta, essential for maintaining brain homeostasis (18). In humans, *post-mortem* studies have shown a loss of AQP4 polarization on the astroglia in patients with AD (PwAD), suggesting impaired glymphatic function (19). The glymphatic system has also been implicated in the pathogenesis of PD, an ND histopathologically characterized by the accumulation of alpha-synuclein (aSyn) aggregates in the brain (20). aSyn is a protein typically found in the brain, but in patients with PD (PwPD), it forms aggregates believed to contribute to dopaminergic cell death (20). Similar to amyloid-beta, studies have shown that exogenously administered aSyn can be cleared from the ISF via the glymphatic system (21). Studies in mice have shown that the infusion of alpha-synuclein into the brain results in its clearance via the glymphatic system, and this clearance is reduced in mice lacking AQP4 channels (22). Interestingly, the proper functioning of the glymphatic system has also been linked to other pathophysiological hallmarks of PD, such as mitochondrial dysfunction (23). Here, bioenergetic disturbances can drive increased protein deposition and subsequent breakdown of the glymphatic system (9). Enhancing our understanding of the glymphatic system could hold the potential for developing multidirectional therapies in patients with ND (PwND) (20). HD, caused by a mutant CAG-triplet repeat expansion in the *HTT* gene, is another ND in which the glymphatic system may play a role (14, 15). The link between the glymphatic system and NDs has significant implications for our understanding of these conditions and their treatment (1, 10, 24). By deepening our understanding of the glymphatic system and its role in NDs, we may be able to develop new therapeutic strategies aimed at improving glymphatic function. These strategies could slow the progression of NDs and increase the quality of life for affected individuals (1, 10, 24). Further research into this topic and its respective role in the onset and progression of NDs could provide new insights into the underlying biology of these debilitating conditions.

### 1.4 Neuroimaging of the glymphatic system

Neuroimaging has emerged as a powerful tool for studying the glymphatic system in health and disease (25). Several techniques have been used to visualize and quantify glymphatic flow *in vivo*, providing unprecedented insights into the function of the glymphatic system and opening up new avenues for diagnosing and treating PwND (26). Positron emission tomography (PET) and magnetic resonance imaging (MRI) are the most commonly used imaging modalities for studying the glymphatic system (7, 25). Imaging techniques from these modalities allow visualization of the glymphatic pathways and quantification of the clearance of solutes from the brain, thus providing valuable insights into possible coping mechanisms in PwND (7, 25). The potential of neuroimaging in establishing new treatment options for PwND is immense (27–29). Visualizing and quantifying the glymphatic system *in vivo* can provide an earlier diagnosis of conditions associated with glymphatic dysfunction. Earlier diagnosis is crucial for effectively managing these conditions, as it allows for the timely initiation of disease-modifying treatments as soon as they become available (27–29). Furthermore, neuroimaging can provide a means to measure the effectiveness of treatments to enhance glymphatic function (6, 10, 24). For instance, drugs that enhance the role of the AQP4 water channels could improve glymphatic clearance and may slow the progression of PwND. Here, neuroimaging can be used to measure the effect of these drugs on glymphatic function, providing valuable information on their efficacy and helping to guide treatment decisions (6, 10, 24). In addition to guiding the development and evaluation of new treatments, neuroimaging of the glymphatic system could also contribute to personalized treatment strategies for PwND. Given the clinical heterogeneity and the variability in glymphatic function between PwND, treatments that enhance glymphatic function may be equally effective only in some patients (30, 31). Here, neuroimaging can provide information on an individual’s glymphatic function, allowing for the personalization of treatment strategies based on this information.

Moreover, neuroimaging could offer insights into the mechanisms underlying the variability in disease progression observed in many PwNDs (26, 32). For instance, PwAD with a similar amyloid-beta burden in the brain (i.e., measured by PET imaging) can exhibit significant variability in cognitive decline (33, 34). This heterogeneity could be partially related to differences in glymphatic function, with impaired glymphatic clearance of amyloid-beta contributing to more rapid cognitive decline (35). Neuroimaging of the glymphatic system could provide insights into these mechanisms, potentially leading to the development of new therapeutic strategies to enhance glymphatic function and slow cognitive decline (6, 10, 24). In conclusion, neuroimaging of the glymphatic system holds significant potential for advancing our understanding of NDs. The latter could lead to the development of individualized treatment strategies. As our knowledge of the glymphatic system continuously expands, so does the potential of pathophysiology-targeted neuroimaging to transform the diagnosis and treatment of PwND.

### 1.5 The scope of this review

In this narrative review, we will focus on the significant role of advanced neuroimaging in studying the glymphatic system in NDs, with particular emphasis on AD and PD, and some recent insights into HD. We will explore the potential of MRI-based neuroimaging techniques in visualizing and quantifying glymphatic flow, providing insights into the function of the glymphatic system in health and NDs. Furthermore, we will discuss how these advanced neuroimaging techniques can guide the development of new, personalized treatment strategies and contribute to early diagnosis, potentially transforming the management of PwND.

## 2 Advanced neuroimaging methods

### 2.1 Motion-sensitizing pulsed field gradient based methods

By using a pair of compensating PFGs, MRI methods can be sensitized to motion by taking advantage of the signal loss due to a loss of phase coherence in a voxel. With a gradient of small length and strength (typical order 10-20 s/mm^2^ described by so-called diffusion-weighting b-factors or b-values), it is possible to measure a phase difference proportional to blood velocity when encoding along a vessel and calculate the volume flow rate (coherent motion in one direction, macroscopic) (36). Recent approaches also use phase contrast MRI to measure CBF (37). Diffusion-weighted imaging (DWI) utilizes higher gradient strengths (b > 100 s/mm^2^), where the flowing signals no longer contribute, to measure water self-diffusion (random molecular motion, microscopic). Application of PFGs also causes signal loss when flow within a voxel is randomized with respect to the orientation of vessels or other water-containing channels. The contribution of this process, named intravoxel incoherent motion (IVIM), is described by a pseudodiffusion constant, D* (38, 39). To assess this type of effect in the brain, a series of b-values of 20 – 100 s/mm^2^ followed by higher values in the diffusion-sensitive range are applied. Both the diffusion constant D and D* can then be obtained by bi-exponential fitting of the data. Since the diffusion *in vivo* is not random but affected by tissue boundaries, these constants are also called ADC and ADC* (apparent diffusion constants or diffusivities). When using DWI with at least 6 different diffusion gradient orientations over a sphere, one can assess the diffusion tensor of water averaged over a voxel, so-called diffusion tensor imaging (DTI), providing diffusion constants along the brain fibers and the fiber orientation. Investigators using these DWI pulse sequences to assess the glymphatic system may tend to call the effects diffusion, but depending on the choice of b-values, all the above-described phenomena can contribute. For instance, when performing standard DWI on a clinical system, it is common to acquire a reference b-value (b_0_) of 0 s/mm^2^ together with one high b-value (800 s/mm^2^) and evaluate the signal loss between them. Therefore, when the literature indicates the use of standard DWI to assess the glymphatic system, all contributions are included, and the apparent diffusion constant (ADC) in the tissue would be affected by the presence of a glymphatic system. Thus, PFG pulse sequences can be a potential tool for assessing the glymphatic system when studying the effects of sub-voxel CSF and ISF spaces in the brain. Key advantages include complete non-invasiveness and wide availability, including the corresponding data analysis software on clinical MR systems. On the other hand, since water molecules can be present in various spaces in the brain, such as macroscopic and microscopic CSF spaces, ISF, and, of course, intracellular space and blood, the signal origins in diffusion-based glymphatic studies are often nonspecific. Thus, care should be taken when interpreting the results.

Long-TE DWI methods with lower b-values have been proposed to measure flow in the perivascular space surrounding large vessels, such as the middle cerebral artery (MCA) (40, 41). More recently, dynamic DWI (dDWI), in combination with an analytical framework, was proposed to measure pulsatile CSF waveforms in the arterial perivascular space by employing a low b-value (150 s/mm^2^) and by using retrospective cardiac gating data to analyze DWI images (42). A multi-b-value scheme termed *Diffusion ANalysis of fluid DYnamics with Incremental Strength of Motion proving gradient* (DANDYISM) was also developed to measure CSF dynamics using DWI images acquired with multiple b-values from 0 to 1000 s/mm^2^ (43). Cardiac-gated IVIM DWI can also measure CSF pulsatility (44). Using the bi-exponential IVIM model, the slow diffusion coefficient was linked with fluid cellularity, the characteristics, and the composition of cells, whereas the fraction of the fast diffusion index (pseudodiffusion constant) was attributed to CSF circulation. CSF circulation refers to the dynamic movement and flow of CSF throughout the brain’s ventricular system and subarachnoid space, playing a key role in the distribution and clearance of solutes and waste products within the CNS. Later, it was reported that the fraction of fast diffusion from IVIM may indicate that pulsatile CSF flow in the lateral ventricles is both direction-dependent and cardiac-dependent (45). From a technical point of view, the term dDWI is used very freely in the literature as it includes intravoxel incoherent motion (which is not diffusion) and bulk flow. This terminology should probably be made more specific in future literature.

A DTI method that has been proposed to assess the glymphatic system is the DTI analysis along the perivascular space (DTI−ALPS) approach (46). This approach hypothesizes to measure the water contributions of motion in the perivascular space as a change in the diffusion constant (diffusivity), with a decreased diffusivity of water indicating dysfunction of the glymphatic system. A fundamental assumption in this method is that the perivascular space is perpendicular to white matter fibers adjacent to the lateral ventricle body. Several studies have used DTI-ALPS to assess the glymphatic system in PwAD and PwPD (30, 46–49). All studies found a lower ALPS index in PwND compared to healthy controls (HCs), interpreted as reduced water diffusivity within the perivascular space. Some studies reported correlations between the ALPS index and amyloid-beta and tau deposition, neuroinflammation, cognitive functions, gray matter integrity, and other conventional neuroimaging markers (30, 46–49). DTI-APLS have also been used to investigate the relationship between the glymphatic system and iron deposition in the normal brain. Zhou et al. applied the DTI-APLS method on 213 healthy volunteers and found that the regional iron deposition, obtained using quantitative susceptibility mapping (QSM), significantly correlated with the APLS index (50). They also found that the APLS index decreased significantly with age, suggesting that the glymphatic system gets impaired with normal aging. The DTI-ALPS method is straightforward since the analysis method can be performed on standard DTI data or already collected DTI data. On the other hand, APLS has been criticized based on the fact that contributions of the perivascular space in white matter should be small to negligible based on knowledge from histology (51). Thus, the exciting results from the literature mentioned above, which have improved our understanding of various diseases, lead us to the question of whether the ALPS index can be a specific marker for certain sub-groups of brain diseases or it may reflect a common change in the brain in all these diseases that have distinct pathology and etiology.

In summary, PFG approaches, which sensitize the experiment to self-diffusion, intravoxel incoherent motion, and bulk flow measurements, have the potential to be valuable tools for studying the human glymphatic system. Further work is merited, especially to validate and disentangle the various signal sources and compartments measured in diffusion MRI to improve their specificity in imaging the glymphatic system in the human brain.

### 2.2 Dynamic gadolinium-based methods

Dynamic gadolinium (Gd)-based MRI methods have historically been used to measure hemodynamic estimates such as, e.g., CBF and cerebral blood volume (CBV). However, these methods have also been implemented to evaluate the circulation of the glymphatic system (52–57). Although the intact BBB prevents Gd from leaking into healthy tissue, the dura blood vessels, having no BBB, will allow Gd to permeate the vessel and subsequently enter the CSF. Thus, post-contrast images often show Gd-induced signal changes in the CSF (58).

The two major dynamic Gd-based methods that are used for measurements in the brain are

i) T_1_-weighted dynamic contrast-enhanced (DCE) MRI, relying on the theory of diffusible tracers (59, 60) and
ii) T_2_^*^-weighted dynamic susceptibility contrast (DSC) MRI-based on the theory of intravascular tracers,

with the latter being the most widespread clinically (61–63). For quantification, both methods usually first convert the acquired signal to concentration and then employ a mathematical approach to the concentration data to derive parameters. In DCE MRI, vascular permeability and interstitial volume and/or plasma volume are usually of interest. In the context of glymphatic system research, the relationship between vascular permeability and interstitial or plasma volume is pivotal, as these factors influence the exchange of fluids and solutes between blood vessels and brain tissue, thereby affecting the efficiency of the glymphatic system’s waste clearance functions. For DSC-MRI, the focus lies on quantifying CBF, CBV, and mean transit time (MTT), parameters that also play an integral role in the functioning of the glymphatic system, as they are key determinants of the brain’s hemodynamic state. Thus, any variations can significantly impact the rate of cerebrospinal fluid (CSF) and interstitial fluid (ISF) exchange, e.g., by influencing the perfusion and respective pressure dynamics. Thus, any variation in these parameters can significantly impact the rate of cerebrospinal fluid (CSF) and interstitial fluid (ISF) exchange, e.g., by influencing the perfusion and respective pressure dynamics (64–66).

**Figure 2:**
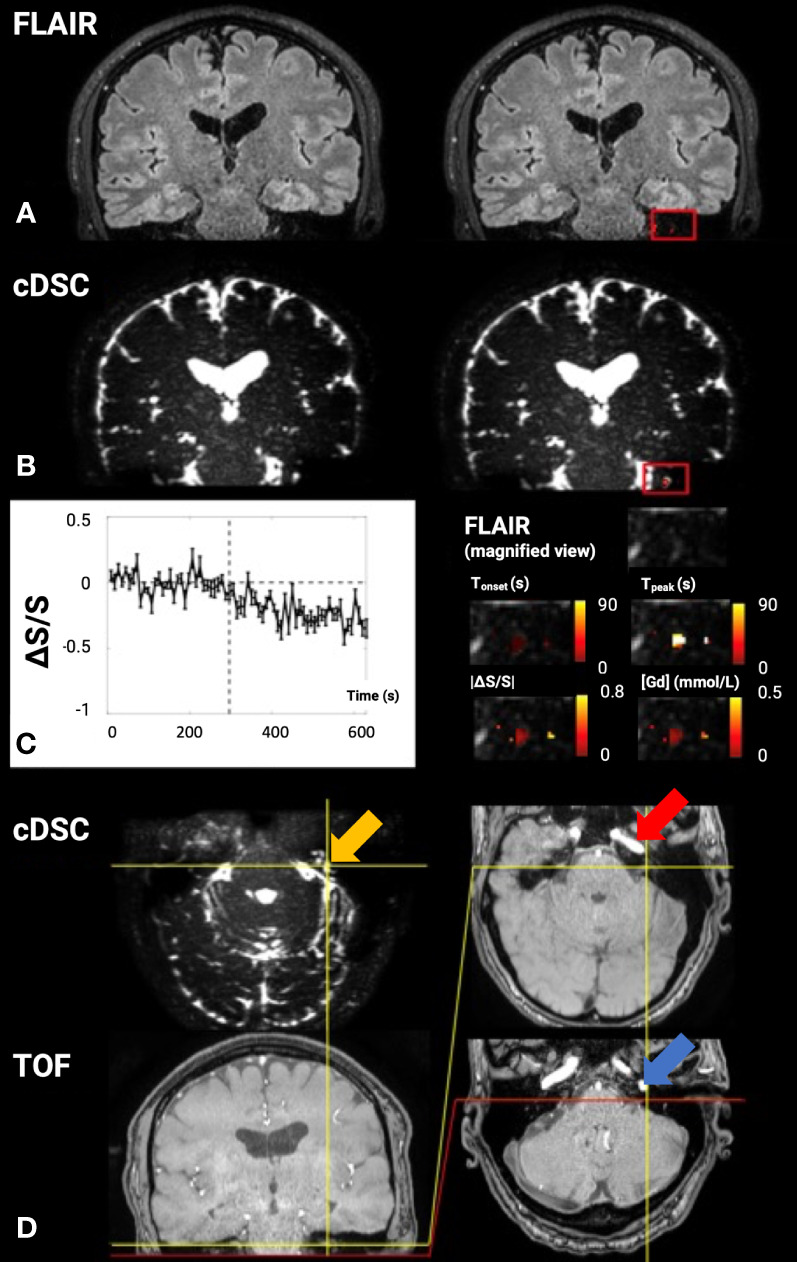
Dynamic gadolinium (Gd)-induced signal changes in the cerebrospinal fluid (CSF) detected by dynamic susceptibility contrast in CSF magnetic resonance imaging (cDSC-MRI) in a 51-year-old healthy volunteer. The signal changes were measured in a small region of interest (ROI) in the brain close to the basal part of the skull, which is considered a major CSF drainage route from the brain and may contain cerebral lymphatic vessels. **(A)** The 3D fluid-attenuated inversion recovery (FLAIR) image is shown as a reference. Relative signal changes (ΔS/S) detected with the 3D FLAIR sequence in the ROI overlaid on the corresponding FLAIR image are shown on the right. **(B)** The cDSC image is shown on the left. Relative signal changes (ΔS/S) detected with cDSC MRI in the ROI overlaid on the corresponding cDSC image are shown on the left. **(C)** The average time course detected using cDSC MRI from the ROI is shown on the left. The error bars indicate standard deviations. The vertical dashed lines indicate the time when Gd is injected. The right panel shows a magnified view of the FLAIR image in the ROI and four maps of the following parameters extracted from the dynamic time courses detected in cDSC overlaid on the FLAIR image: T_onset_, onset time; T_peak_, time to peak, absolute value of relative signal change |ΔS/S| between pre- and post-Gd, [Gd] = concentration of Gd. The color bars indicate the corresponding scales of each parameter. **(D)** Additional cDSC and TOF-MRA (time-of-flight magnetic resonance angiography) images (coronal view on the bottom left and axial views on the right) are shown to confirm the anatomical location of the ROI. The yellow arrow indicates the location of the highlighted and expanded voxels. The red arrow points to the petrous internal carotid canal with a high flow signal on the TOF sequence within the petrous internal carotid artery (ICA). The posterior portion of the petrous ICA makes a ~90° inferior turn to exit the petrous carotid canal at the inferior skull base. The carotid foramen (which is a bone structure and should appear dark on both cDSC and TOF images) is located at the posterior end of the petrous ICA, which can be identified on the TOF images (blue arrow). Although the jugular foramen (also a bone structure that should appear dark on both cDSC and TOF images) cannot be directly identified in the current images, it should be located immediately posterior to the carotid foramen. Therefore, the highlighted voxels in the ROI in **(C)** should be located above the carotid foramen and jugular foramen inside the skull (75).

The first dynamic-Gd studies used DCE MRI in a preclinical setting, and the acquired data were usually evaluated using semi-quantitative methods, such as assessing signal change relative to the baseline (52–54, 57). These studies were followed by human DCE MRI studies (67, 68) and studies utilizing tracer kinetic models for analyzing the DCE MRI data (53, 69). In the pre-clinical studies, the Gd-contrast agent was administered intrathecally with intra-cisterna magna injection, which offers a direct and accurate assessment of the glymphatic system. In the human studies, intravenous and intrathecal injection was used. To our knowledge, intra-cisterna magna injection, which is more invasive than intravenous intrathecal injection, has not been used in human ND research. Since Gd-contrast agents are not FDA-approved for intrathecal administration in humans, some studies have instead used intravenous contrast agent administration (70–74). **Figure 2** shows an example from one of these studies. Several methods have been developed to measure intravenous Gd-contrast-induced signal changes in the CSF (i.e., as reviewed by Verheggen et al., 2021) (76). In addition, it has also been shown that it is possible to measure Gd-induced signal changes in both the blood and lymphatic vessels in the human brain using DSC in a single scan (56).

Two important caveats should be noted when using Gd-based methods to investigate the glymphatic system. First, depending on the pulse sequence and imaging parameters applied, hyperintensities or high post-Gd signal changes do not always correspond to higher Gd concentration (55, 77). Second, substantial partial volume effects from the blood compartment should be considered when interpreting the Gd-induced signal changes in the brain. Based on previous work, T2-weighted spin echo sequences with a very long TE (> 600 ms) may be the best choice to minimize these confounding effects by selecting CSF signal only (55, 56).

One of the predominant diseases for which Gd-based contrast agents are used to study the glymphatic system is AD. Harrison et al. (78) compared DCE MRI in mice with tauopathy with controls and found that CSF-ISF exchange and clearance became impaired in the caudal cortex of the tauopathy mice. Ben-Nejma et al., used DCE to study mice with amyloid-beta deposition (79), revealing significantly reduced glymphatic flow in the diseased mice compared to the controls, which was interpreted in terms of reduced and redirected flow. DCE MRI studies have also been performed to study the effects of treatment. Alghanimy et al. observed that glymphatic transport was significantly enhanced in healthy rats when an AQP4 facilitator was used (80). Other dementias have also been investigated. In a study by Ringstad et al., patients with normal pressure hydrocephalus (iNPH)-related dementia were compared to HCs using DCE MRI (67). It was revealed that the patients with iNPH-associated dementia had a delayed clearance compared to the healthy controls (67).

It has been suggested that meningeal lymphatic vessels facilitate the drainage of CSF and ISF, acting as an exit route for the glymphatic flow. Thus, an impairment of the meningeal lymphatic drainage may aggravate α-syn pathology and exacerbate glymphatic clearance deficits in PD (81). The study by Ding et al. found that patients with idiopathic PD had significantly reduced flow through the meningeal lymphatic vessels along the superior sagittal sinus and sigmoid sinus compared to patients with atypical Parkinsonian disorders (PwAP) (82). DCE MRI may thus be used to differentiate PwPD from PwAP, which is often challenging, especially in the early stages of these diseases (83). In summary, dynamic Gd approaches are still considered the gold standard for studying the glymphatic system in humans. Intrathecal administration of the Gd contrast can provide a direct assessment of CSF circulation in the brain. Nevertheless, the intrathecal procedure is more invasive and is not FDA-approved for human use. Therefore, although they are invasive and have relatively long acquisition times, methods based on intravenous Gd injection may have more translational value for routine clinical use.

### 2.3 Dynamic glucose-enhanced methods

Dynamic glucose-enhanced (DGE) MRI is a relatively new contrast-enhanced imaging technique that uses natural sugar (d-glucose) or sugar analogues together with dynamic Chemical Exchange Saturation Transfer (CEST) imaging to obtain information about glucose delivery, tissue transport, and metabolism (84). When selectively radiofrequency-labeling the hydroxyl protons in D-glucose, the continuous exchange with unsaturated water protons will create a measurable reduction of the MRI water signal (85, 86). Thus, using the CEST technique, the millimolar concentration of D-glucose can be detected through a reduction (saturation) of the signal from water (present at molar concentration), facilitating an approach called glucoCEST (87–94). The advantage of D-glucose as a contrast agent is that it is affordable and has widespread availability. It is also a regulatory-approved biocompatible substance already used in glucose tolerance testing of diabetics in the clinic. However, DGE imaging produces a small effect size (a few percent, i.e., on the order of functional MRI signal changes), especially at clinical magnetic field strengths (1.5T and 3T) and long scan durations (>10 min) are required due to the large amount of D-glucose solution needed. This makes the technique vulnerable to patient motion, which can produce signal changes of the same order of magnitude as the true CEST signals, so-called pseudo-CEST effects (95, 96). Similar to DSC- and DCE-MRI, DGE-MRI also measures a dynamic tissue response curve. While continuously saturating OH protons at one chosen frequency offset, signals are acquired dynamically at baseline, during and after infusion. The D-glucose is injected intravenously after a pre-set time (a few minutes), i.e., when sufficient baseline images have been acquired. The resulting DGE tissue response curve is then obtained by subtracting the average of pre-injection scans from the post-injection scans at each time point. The resulting DGE MRI curve provides insights into the changes in D-glucose concentration within tissues.

**Figure 3:**
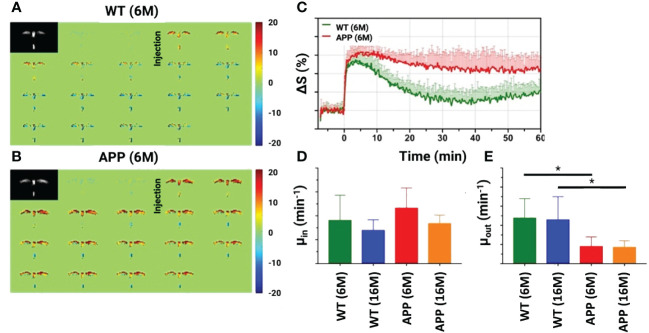
Dynamic glucose-enhanced magnetic resonance imaging (DGE-MRI) results for the cerebrospinal fluid (CSF) in both an Alzheimer’s disease mice model (APP/PS1) and wild-type (WT) mice. Dynamic difference images before and after infusion for WT **(A)** and APP/PS1 **(B)** mice at 6 months (6M). **(C)** Experimental (solid line) and fitted (dashed line) CSF DGE curves for WT (6M, n=5) and APP/PS1 (6M, n=5) mice. Comparison of fitted uptake and clearance rates μ_in_ **(D)** μ_out_ **(E)** between WT and APP/PS1 mice for two age groups (6M and 16M). *: p<0.05. Reproduced with permission from J. Xu (98). .

The high concentrations of glucose transporters in brain capillary endothelial cells result in D-glucose traversing both the BBB and the blood-CSF barrier (BCSFB). This unique characteristic allows the DGE method to investigate the integrity of BBB and the CSF exchange process with parenchyma, i.e., the CSF-ISF exchange. Studies by Huang et al. and Chen et al. demonstrate that DGE MRI can concurrently assess glucose transporter and glymphatic system functioning in AD (97, 98). Both studies used DGE to detect glucose in the CSF and parenchyma. The study by Chen et al. scanned a mouse model with tauopathy at 7–8 months of age at 11.7T. It was found that D-glucose uptake in parenchyma and CSF was reduced compared to WT mice. Additionally, a slower D-glucose uptake rate was observed in the CSF of the tau mice in comparison to their WT counterparts, indicating the presence of impaired glucose transporters at both the BBB and the BCSFB in these tau mice (97). In the paper by Huang et al., AD mice with amyloid plaques (APP/PS1) were age-matched with wild-type (WT) mice and scanned at 3T. The D-glucose uptake and clearance in CSF of the APP group are illustrated in **Figure 3**. D-glucose clearance was threefold lower in the young (6-month-old) AD mice than in WT mice. Old mice (16 months old) also showed reduced clearance. Further, compared to young AD mice, the maximum signal of D-glucose uptake was lower in old AD mice. The findings in this study indicate that DGE MRI can identify changes in both glucose uptake and clearance in AD, even at an early stage of the disease (98). A very recent study used DGE to measure D-glucose clearance from CSF in an HD mouse model (Q175 knock-in with abnormal accumulation of mHTT: mutant toxic Huntingtin protein), showing impaired clearance already in premanifest mice and worsening as the disease progressed (16). Interestingly, these investigators also studied the expression of the AQP4 channels in the perivascular compartment, which was significantly diminished in the HD mouse brain. Due to the novelty of DGE MRI, no studies on PwNDs have been published yet. In summary, the dynamic glucose-enhanced method seems to have potential when assessing the glymphatic system in humans. However, further work is merited, especially when moving towards patient studies. For example, the small effect size should be addressed together with the confounding effects that appear due to motion. In addition, studies on other neurodegenerative diseases are warranted.

### 2.4 Other methods

This review has focused on some of the MRI-based methodologies currently being applied for the study of PD, HD, and AD. However, the field is young, and other MRI-based approaches with the potential to image the glymphatic system and its properties continue to be developed. This includes methods such as Phase Contrast MRI (99), Spatial modulation of magnetization (SPAMM) (100), Time-spatial labeling inversion pulse (Time-SLIP) (101), arterial spin labeling (ASL) (102), and ultra-fast magnetic resonance encephalography (103). These noninvasive approaches can be added easily to a clinical MRI exam and will no doubt be applied to ND in the near future.

## 3 Conclusion

In recent years, exploring the glymphatic system using advanced MRI techniques has provided valuable insights into its role in NDs. This narrative review has focused on three critical MRI-based methodologies: intravoxel-incoherent motion, DCE/DSC, and DGE methods, shedding light on the exciting progress in understanding the glymphatic system’s involvement in these diseases. The accumulating evidence from both preclinical models and clinical studies strongly supports the hypothesis that glymphatic dysfunction plays a pivotal role in the pathogenesis of NDs, including AD, PD, and HD. The glymphatic system’s function in clearing toxic proteins is paramount, as impaired clearance mechanisms may contribute to their accumulation. Therefore, understanding the glymphatic system’s role in these disorders opens new possibilities for establishing novel progression markers, a crucial prerequisite for conceptualizing clinical trials investigating the disease-modifying properties of drug candidates. The non-invasive nature of these MRI techniques allows for longitudinal studies in both preclinical models and human subjects, enabling researchers to track changes in glymphatic function over time. In addition, pharmacologically enhancing glymphatic clearance represents a novel approach to mitigating the progression of NDs. By promoting the efficient removal of toxic proteins and waste products from the brain, we may be able to slow down or even halt the neurodegenerative processes. Future research should focus on developing and testing compounds that can modulate glymphatic function safely and effectively. Treatment monitoring using the proposed neuroimaging methods will inform noninvasively on the success or failure of these endeavors, likely speeding up the development of new drugs.

As we look to the future, several important avenues for research become evident. Firstly, further studies are needed to elucidate the mechanisms underlying glymphatic dysfunction in neurodegenerative disorders. It would be crucial to enhance the interpretability of IVIM-based methods or refocus on the other proposed methods. Here, DGE could provide future insights while relying on a well-tolerated and FDA-approved D-glucose infusion. However, studies in PwNDs are currently sparse, and real-world evidence is needed to validate this method in a clinical setting. Moreover, expanding the application of advanced neuroimaging techniques to more extensive and diverse patient populations will provide a broader understanding of the glymphatic system’s role in different stages and subtypes of neurodegenerative diseases. Additionally, developing more sensitive and specific imaging markers for glymphatic function could improve the accuracy of diagnosis and monitoring of these disorders.

In conclusion, the glymphatic system is a fascinating and essential component of brain health, and its dysfunction appears to be intricately linked to the pathogenesis of NDs. Developing advanced neuroimaging techniques has been instrumental in advancing our knowledge in this field. With continued research and collaboration, we can harness this understanding to develop innovative therapies that target the glymphatic system, potentially changing the trajectory of PwND. The future holds promise for unlocking the full therapeutic potential of the glymphatic system and ushering in a new era of individualized treatments for PwNDs.

## Author contributions

JP: Conceptualization, Writing – original draft, Writing – review & editing. JX: Conceptualization, Writing – original draft, Writing – review & editing. JH: Conceptualization, Writing – original draft, Writing – review & editing. PvZ: Conceptualization, Writing – original draft, Writing – review & editing. LK: Conceptualization, Writing – original draft, Writing – review & editing.
